# PEER (DYADIC) SUPPORT: EXPERIENCES AND PERSPECTIVES FROM OLDER AFRICAN AMERICAN WOMEN WITH HYPERTENSION

**DOI:** 10.1093/geroni/igae098.0889

**Published:** 2024-12-31

**Authors:** Angela Groves, Julie Ober Allen

**Affiliations:** Western Michigan University, Kalamazoo, Michigan, United States; University of Wisconsin, Madison, Wisconsin, United States

## Abstract

African American women have a higher prevalence of hypertension than women of other ethnicities. Interventions have not adequately addressed the higher prevalence of hypertension in this population. Social support networks can be an effective strategy for improving hypertension self-management behaviors. The objective of this study was to evaluate the feasibility of an 8-week peer (dyadic) support intervention to improve Dietary Approaches to Stopping Hypertension (DASH) adherence and reduce systolic blood pressure among older African American women with hypertension and describe the relationship that occurred during the intervention. This mixed methods study used a convergent parallel design. A total of 40 African American women diagnosed with hypertension, aged 60 and older were paired to form 20 dyads. At the end of the 8-week intervention, participants discussed their experiences in a 60-minute focus group session. Content analysis was used to analyze focus group interviews. Qualitative preliminary data analysis will be presented. Emerging themes included, accountability, epiphany, partnership-dyad, communication, and receiving support. Preliminary findings suggest that peer (dyadic) support is feasible and can be an effective intervention strategy to improve DASH diet and blood pressure management. Future studies should examine the extent in which dyadic relationships are maintained over time to improve diet and blood pressure outcomes. Also, future randomized controlled trials should be conducted to evaluate the effectiveness of the peer dyadic support modality on DASH diet adherence to reduce systolic blood pressure.

